# Using Korean Dramas as a Precision Mental Health Education Tool for Asian Americans: A Pilot Study

**DOI:** 10.3390/ijerph16122151

**Published:** 2019-06-18

**Authors:** Van My Ta Park, R. Henry Olaisen, Quyen Vuong, Lisa G. Rosas, Mildred K. Cho

**Affiliations:** 1 Department of Community Health Systems, School of Nursing, University of California at San Francisco, San Francisco, CA 94143, USA; 2 Department of Population and Quantitative Health Sciences, Case Western Reserve University, Cleveland, OH 44106, USA; rho2@case.edu; 3 International Children Assistance Network, Milpitas, CA 95035, USA; quyen.vuong@ican2.org; 4 Department of Health Research and Policy, Stanford University, Stanford, CA 94305, USA; lgrosas@stanford.edu; 5 Department of Primary Care and Population Health, Stanford University, Stanford, CA 94305, USA; micho@stanford.edu; 6 Center for Biomedical Ethics, Stanford University, Stanford, CA 94305, USA

**Keywords:** Asian Americans, precision mental health, Korean dramas, community health, health education, health disparities

## Abstract

Precision mental health (MH) holds great potential for revolutionizing MH care and reducing the burden of mental illness. Efforts to engage Asian Americans in precision MH research is necessary to help reduce MH disparities. Korean drama (“K-drama”) television shows may be an effective educational tool to increase precision MH knowledge, attitudes, and behaviors (KAB) among Asian Americans. This study determined whether KAB improved after participating in a K-drama precision MH workshop, and examined the participants’ perspectives about K-dramas’ utility as an educational tool. A K-drama precision MH workshop in English/Vietnamese/Korean was conducted with a convenience sample (*n* = 122). Pre-/post-tests on precision MH KAB (genetics and genetic testing, and MH and help-seeking) and a survey on K-dramas’ utility as an educational tool were administered. Findings revealed a significant difference in the pre- and post-test KAB scores overall, by genetics and genetic testing, and by MH and help-seeking. There were also significant increases in the overall post-test KAB scores by workshop (language) participation. Overall, participants responded positively on the utility of K-dramas as a precision MH educational tool. This study demonstrates the feasibility of K-drama as an innovative and widely available health education tool to educate communities about precision MH.

## 1. Introduction

Precision health (also known as “precision medicine”) has been defined as, “an innovative approach that takes into account individual differences in people’s genes, environments, and lifestyles” [1]. Researchers seek to use precision health as an approach to preventing, predicting, and treating disease [2]. In recent years, precision health has gained national prominence. Spearheaded by the National Institutes of Health, the Precision Medicine Initiative (PMI) was launched to “leverage advances in genomics, emerging methods for managing and analyzing large datasets while protecting privacy, and health information technology to accelerate biomedical discoveries” [3]. In December 2016, the 21st Century Cures Act was passed which authorized a total of $1.5 billion over the next ten years for the *All of Us* research program, which will recruit more than one million volunteers in the United States (US) to help achieve the goals of the PMI [3,4]. 

Recently, there has been increased attention paid to how precision health is relevant for mental health (also known as “precision mental health”). Precision mental health is defined as “an approach to prevention and intervention that focuses on obtaining an accurate understanding of the needs, preferences, and prognostic possibilities for any given individual… and which tailors interventions and support accordingly in line with the most up-to-date scientific evidence” [5]. Precision mental health is an imperative area of research, especially due to the global burden of mental illnesses. For example, more than 300 million people of all ages suffer from depression making it the leading cause of disability worldwide [6]. Although there are effective psychological and medical treatments available, many depression cases are left untreated [6]. Moreover, treatment is typically provided on a “trial and error” approach which means that many patients may suffer for years and are susceptible to chronic disability, treatment resistance, and suicide [7]. In addition, Asian Americans often experiment with medications (e.g., antidepressants) due to the belief that such medications may be “too strong for their constitution” [8]. A number of Asian Americans may also use herbal treatments, which may then result in unintended drug interactions [8]. Furthermore, data from six East Asian countries revealed that for Asian patients with major depressive disorder, reasons for discontinuation of pharmacological treatments include inadequate response (64.1%) and adverse events (4.1%) [9].

Given the massive amount of money dedicated to precision health in the US [10] and the potential to revolutionize mental health care and reduce the mental health disability burden through precision mental health [5], it is imperative that all persons, including racial and ethnic minority populations in the US, are meaningfully included in precision mental health efforts and research. If racial and ethnic minorities are not included in precision health and precision mental health, health disparities would likely be exacerbated. In particular, it is important to ensure that underserved communities, such as Asian Americans, are included in precision mental health strategies as Asian Americans comprise of 6% of the US population, and are the fastest growing racial group in the US [11]. However, addressing mental health needs of Asian Americans is challenging because they are heterogeneous in regards to languages and dialects, cultural groups, immigration patterns, religions, diets and socio-economic status [11]. There are significant health and health care disparities among Asian Americans. For example, Asian Americans tend to underuse mental health services even though there is a demonstrated need [12] (i.e., Southeast Asians have high rates of post-traumatic stress disorder (PTSD) [13,14]). Asian American culture is characterized as a collective culture, where strong themes of familial hierarchy, honor of family name, and healing and support from within family and close friends, are present [15]. These cultural patterns influence perceptions and biases about mental health and its treatments [16]. In tune with their collective culture, Asian Americans often prefer to use social support networks, familial ties, indigenous healers, and religious/spiritual outlets, to treat psychological or somatic symptoms [17], rather than seek professional help. Multiple research findings suggest Asian Americans underuse mental health services even though they show similar depressive symptoms [18,19,20].

Given the US’s relatively recent priority on precision health, Asian Americans’ unique perceptions and experiences about mental health, and the need for mental health education and outreach that recognizes cultural heterogeneity, this study sought to engage and educate Asian Americans about precision mental health in a culturally appropriate and innovative way. Specifically, we used Korean drama (a.k.a. “K-drama”) television shows to educate Asian Americans about precision mental health including genetics and genetic testing as well as mental health and help-seeking among English-, Vietnamese-, and Korean-speaking Asian Americans. Thus, the exploratory study’s objectives were (1) to determine whether the participants’ knowledge, attitudes, and behaviors (KAB) improved after participating in a K-drama precision mental health workshop, and, (2) to examine the participants’ perspectives about the utility of K-dramas as a precision mental health educational tool.

K-dramas have global popularity among all ages and races, including Asian Americans [21]. K-dramas are short (e.g., 2 episodes per week over 2 months), translated in numerous languages, watched in about 100 countries, and widely available online including on Hulu, Netflix, and YouTube [21,22,23]. Research has shown that K-dramas are uniquely popular in many cultural groups, and have created meaningful cultural changes (e.g., positively affected Japan’s attitudes about ethnic Koreans’ identity and social position in Japan) [21]. Furthermore, K-dramas may play a significant social-marketing tool given their intense emotional plots in conjunction with their worldwide prominence [22,24,25]. K-dramas are different from US television shows in the lack of sexual content as well as its focus on Confucian values such as responsibility and respect [22]. K-dramas are exported and broadcasted all over the world [26,27]. The US also has a substantial following of K-drama with about 18 million Americans [23,24]. A substantial portion of viewers is non-Korean-speaking in the US, and even includes native Spanish speakers [22,28].

To the authors’ knowledge, no research has been conducted or social-marketing program exists that uses and examines the feasibility of K-drama, an innovative, non-invasive, low-cost, and widely available social-marketing and population-based tool/medium to educate communities, including Asian Americans, about precision mental health.

## 2. Materials and Methods 

### 2.1. Design and Methods

A community-based sample of participants was recruited to participate in this cross-sectional study through local community organizations that serve Asian Americans, Vietnamese-language radio, flyers, word of mouth, and social media (e.g., Facebook). Trained bilingual and bicultural Vietnamese and Korean American research staff screened potential participants to determine their eligibility. Prior to the start of the 90-min K-drama workshops, participants signed an informed consent, completed a socio-demographic questionnaire, and a pre-test survey on their KAB about precision mental health. At the conclusion of the workshops, participants completed a post-test KAB precision mental health survey and a survey about their perspectives on the utility of K-drama. All participants were informed that the purpose of this study was to examine the use of K-dramas to improve the KAB on precision mental health. Participants signed an informed consent to participate in one of 12 workshops (four in each language group—English, Vietnamese, Korean). These workshops were conducted at four different community sites in the San Francisco Bay Area. Each participant was given a $25 gift card to a local store for his or her time as well as a folder with information on local mental health resources in the respective workshop language of participation.

### 2.2. Participants

The inclusion criteria included: (1) self-identified as Asian American; (2) can read, write, and speak English, Vietnamese or Korean; (3) were at least 18 years old; (4) had ever watched or currently watched K-drama; and, (5) resided in the San Francisco Bay Area. Of the 140 persons who signed up to participate in the study, 18 did not participate due to family emergencies, work conflicts, or other reasons that were not specified. During the screening, potential participants were asked if they self-identified as Asian or Asian American (for English-speaking), Korean or Korean American (for Korean-speaking), Vietnamese or Vietnamese American (for Vietnamese-speaking).

### 2.3. K-drama Precision Mental Health Workshops

Each K-drama workshop had a bilingual (English/Vietnamese; English/Korean) and bicultural (English/Vietnamese; English/Korean) workshop facilitator and at least one other supporting research staff present. There were three facilitators, one for each language (English, Vietnamese, Korean) as the workshops were conducted in these respective languages. The K-drama workshop curriculum comprised of an interactive discussion on health topics including precision health, mental health, and help-seeking, as well as four 6–8 min K-drama scenes that were drawn from three different K-dramas shows. The K-drama shows included (1) *Bubblegum,* (2) *It’s Okay, It’s Love*, and, (3) *Me Too, Flower*, which were released in 2011 to 2015. 

The workshop began with an introduction to the study. Then, a series of discussions centered on specific topics were discussed in the following order:

(a) precision health, genetic testing, mental health, and precision mental health; followed by a K-drama scene on genetic testing for Alzheimer’s disease; followed by a discussion on willingness to get a genetic test;
(b) K-drama scene on depression and treatment, followed by precision mental health discussion;
(c) K-drama scene on patient-provider trust followed by a discussion on willingness to participate in research and patient-provider trust;
(d) K-drama scene on help-seeking and compassion, followed by a discussion on help-seeking and compassion.

All topics were themes of the K-drama scenes, which were pieced together to inform participants about what “precision health” was. After each K-drama scene was shown, the participants’ reactions (i.e., thoughts; feelings) were elicited. A facilitator guided a semi-structured discussion on specific topics and how these related to precision health (e.g., definition of precision health, and its role with mental health). For example, after a K-drama scene on genetic testing for Alzheimer’s disease was shown, participants were asked: *Is the use of personal genetic information in health care beneficial to patients? Why or why not?* In another example, after a K-drama scene on a patient receiving therapy for her depression, participants were asked: *Do you feel comfortable talking with your doctor(s) about mental health? Why or why not?*

### 2.4. Measures

The socio-demographic questionnaire included 19 questions, including gender, age, marital status, education, employment, household income, nativity, and English-language proficiency. 

The KAB pre- and post-test survey comprised of two main categories of items: *Genetics and Genetic Testing*, and *Mental Health and Help-Seeking*. Participants were asked to indicate to what extent they agreed with the statements in the survey on a five-point Likert scale from “strongly disagree” (“1”) to “strongly agree” (“5”). These *Genetics and Genetic Testing* items, which were used in a study that elicited the diverse racial/ethnic minority communities’ perspectives on precision health, are not a standardized instrument [29]. The *Genetics and Genetic Testing* items included: (1) Genetics affect health; (2) The use of personal genetic information in health care is beneficial to patients; (3) I feel comfortable talking to my doctors about genetic testing; (4) The use of genetic tests could lead to discrimination; (5) If available, I would like to undergo genetic testing to find out if I am at risk of certain diseases (e.g., cancer, diabetes, etc.); (6) If available, I would like to undergo genetic testing to find out if a certain drug (e.g., high blood pressure medication) or treatment would work for my conditions; and, (7) If available, I would like to participate in research about using genetics for disease prevention and treatment. 

The *Mental Health and Help-Seeking* items were developed for the purpose of this study and included: (1) I understand what mental health is; (2) I am familiar with some mental health conditions; (3) I believe that stress, worries, and happiness can impact one’s overall health including mental health; (4) I know people who will listen and support me when I need to talk to someone; (5) I feel comfortable sharing my personal experiences with others; (6) I feel comfortable talking to my doctors about my mental health; (7) If needed, I would seek help for mental health reasons including, but not limited to, stress, anxiety, and depression; (8) I know how to get services or community resources if I or my family need them; and, (9) I know how to get help in a crisis.

In addition to the KAB post-test survey, a survey asking about the participants’ perspectives about the *Utility of K-drama on Precision Mental Health* was developed and administered at the conclusion of the workshop. Participants were asked five items and indicated to what extent they agreed with the statements in the survey on a five-point Likert scale from “strongly disagree” (“1”) to “strongly agree” (“5”). Items included: (1) I believe that Korean dramas can help the community to understand how genetics affects health; (2) I believe that Korean dramas can help the community to get genetic testing; (3) I believe that Korean dramas can help the community to understand what mental health is; (4) I believe that Korean dramas can help the community to seek help for mental health problems such as stress, anxiety and depression; and, (5) I am satisfied with the workshop I received.

### 2.5. Translation Process and Validity

The following materials were translated into Korean and Vietnamese: recruitment flyers, screening eligibility questions; informed consent; socio-demographic questionnaire; KAB survey; workshop presentation; and, local information about mental health resources. The World Health Organization’s process of translation and adaptation of instruments [30] was used to guide the translations of the study materials. By using this established translation method such as forward and backward translation methods, we were able to attain “conceptually equivalent” Korean and Vietnamese-language versions of the English materials, which were translated to be “cross-cultural and conceptual, rather than on linguistic/literal equivalence” [30]. Bilingual and bicultural Korean and Vietnamese research staff conducted the translations.

### 2.6. Data Analysis

Descriptive statistics were provided for the overall sample and by workshop participation (language). Bivariate differences of socio-demographic characteristics were assessed and tested for statistical significance using Wald (continuous age) and Chi-square test statistics (categorical variables). Evaluation included pre-post assessment of individual items (x = 16), construct scores (genetics and genetic testing, mental health and help-seeking), and an overall summary score. Summary scores aid with improving evaluability, yielding continuous outcome measures. The summary score took a theoretical value from 1–5 (“strongly disagree” to “strongly agree”), and represented the overall mean score across eligible items. Unweighted and weighted summary scores were generated for the genetics and genetic testing items as well as the mental health and help-seeking items. Overall weighted summary scores took into account that mental health and help-seeking components have two more items than the genetics and genetic test component (weighted towards mental health), while overall, unweighted analysis treated each subscale as equal in contribution to overall score. Genetic item #4, “The use of genetic tests could lead to discrimination,” was reverse coded. Using paired t-tests, we tested for overall mean change in true differences in means, as well as a sample means of the differences. One-way analysis of variance (ANOVA) was employed to test whether the K-drama had an equivalent effect across language groups. To identify which groups if any, performed better, a post-hoc, Tukey’s Honest Significant Difference test was performed. 

Out of the 122 participants, 118 provided complete data on the outcome. Of the four participants with missing outcome data, three participated in the Korean and one in the Vietnamese workshops. Tables 1 and 4 included data from 122 participants except for: (a) household income—three participants declined to report it; and, (b) “enjoy watching K-dramas”—one participant did not answer this question. For number of hours per week watching K-dramas, the *n* was based on their affirmative (“Yes”) response to “currently watches K-dramas” (*n* = 85). Tables 2 and 3 included data from 118 participants. Statistical analyses were performed using R version 3.5 and the Hmisc and tableone packages.

### 2.7. Human Subjects Protection

This research was approved by the Stanford Institutional Review Board (protocol #: 42209). Informed consent was obtained from the participants prior to study participation.

## 3. Results

### 3.1. Sample Characteristics (Table 1) 

A total of 122 persons participated in the workshops including 45 in the Korean-speaking, 36 in the English-speaking, 41 in the Vietnamese-speaking workshops. The English-speaking participants included Chinese (including Taiwanese) *n* = 7, Filipino *n* = 3, Japanese *n* = 4, Burmese *n* = 1, Vietnamese, *n* = 4, Korean *n* = 3, Mixed *n* = 7, and “Asian” unspecified, *n* = 6 (in other words, they wrote “Asian” for their race). The overall sample was mostly female. Workshop participation (language) did not differ by sex or in K-drama behaviors including number of years watching K-dramas, currently watching K-dramas, number of hours per week watching K-dramas, and whether they enjoy watching K-dramas. There were significant workshop participation differences for several sample characteristics including age, marital status, education, employment status, household income, and nativity. The English-language workshop participants tended to be younger, single, and college-educated. The Korean-language workshop participants tended to be older (≥65 years), married/living together, and college-educated. The Vietnamese-language participants tended to be middle-aged, with lower educational attainment than the other two groups, and from low-income households. There were higher proportions of participants in both the Korean and Vietnamese-language workshops who were foreign-born compared to the English-language workshops.

### 3.2. Unweighted and Weighted Pre- and Post-Summary Scores: Overall and by Genetic Testing and Mental Health and Help-Seeking (n = 118) (Table 2)

Overall, for both the unweighted and weighted KAB scores, the mean post-test score was 0.19 points greater than on the pre-test (95% confidence intervals (CIs) ranging from 0.13 to 0.25). For the genetic testing summary score, the mean change was 0.19 points greater at post-test than at pre-test (95% CI: 0.11, 0.28). For the mental health and help-seeking summary score, the mean change was 0.19 points greater at post-test than at pre-test (95% CI: 0.11, 0.26). 

### 3.3. Pre- and Post-Difference in Summary Scores by Socio-Demographic Characteristics: Overall, Genetics and Genetic Testing, and Mental Health and Help-Seeking (Table 3)

Subgroups’ post-scores increased overall, and by the genetic testing and mental health and help-seeking components. Overall, all the characteristics were associated with significantly improved KAB scores at post-test except for participants who were homemakers. For the genetics and genetic testing component, there was no evidence of effect for the following subgroups: homemaker, participants with household incomes of ‘$75–150K’ and ‘>$150K’, and college-educated. For the mental health and help-seeking component, there was no evidence of effect for participants who were 65 years old or older, homemakers, persons who were ‘not looking’ for employment, and Korean-speaking participants. 

### 3.4. Perspectives About the Utility of K-Drama As a Precision Mental Health Educational Tool (Table 4)

Overall, the participants responded positively to all the five items asking about the utility of K-dramas as a precision mental health educational tool (mean scores ranged from 4.09–4.50). There were statistically significant differences by workshop language participation for items 1–4. For example, the Vietnamese-speaking workshop group scored significantly higher than the Korean-speaking workshop group on all four questions. Satisfaction with the intervention was not significantly different by language groups.

## 4. Discussion

### 4.1. Summary of Findings

Our findings suggest that Korean dramas are a promising and feasible tool for providing education about complex and sensitive issues such as genetic testing and mental health. In addition, our study suggests that K-dramas are positively viewed among diverse Asian American communities, across languages and ethnicities. 

The impact of the K-dramas on KAB scores varied for different subgroups, potentially indicating subpopulations for whom K-drama-based tools would be more effective at improving understanding or changing behaviors. For example, some of the largest increases in post-test scores were among those who reported educational attainment levels of high school or less, suggesting that K-dramas might be particularly useful for this group. Similarly, those reporting lower household income levels had larger increases in post-test scores and could be an indication of higher utility of a K-drama approach for this group.

The English-language workshop participants had relatively higher increases in post-test scores while the Korean-language participants had lower increases. This difference between languages could partially be attributed to the differences in age between these workshop participants; most English-language workshop participants were less than 35 years old, while the majority of Korean-language workshop participants were over 65. One hypothesis is that “with age it becomes harder to make behavioral changes, but once those changes are initiated, older adults find them easier to maintain” [31].

While only 58.5% of the Vietnamese-speaking workshop participants reported that they currently watched K-dramas (compared to 82.2% of Korean-speaking and 66.7% of English-speaking workshop participants), a greater proportion of the Vietnamese-speaking workshop participants who currently watched K-dramas (79%) reported that they watched two or more hours of K-dramas per week than their counterparts (70% of Korean-speaking and 50% of English-speaking). It is also interesting to note that the Vietnamese-speaking workshop participants had the highest mean scores in response to the survey on the utility of K-dramas as an educational tool for precision mental health. 

It is possible that there was social desirability bias when Korean participants completed the pre-test survey because this was administered prior to the start of the workshop and they did not want to share how much they understood precision mental health and/or realized after the workshop a more accurate assessment of their KAB on precision mental health.

### 4.2. Strengths and Limitations

Given that this is a pilot study with convenience sampling, the findings may not be generalizable to the population. The cross-sectional nature of the study did not allow for a follow-up with participants. Also, for each language workshop, different recruitment methods were employed which may partially explain the different sample characteristics. For example, two of the four Korean-language workshops were conducted at community organizations that serve older adults. Also, some Vietnamese-language workshop participants heard about the study from the Vietnamese-language media where the listeners tend to be older [32]. The differences in language preference (English vs. Korean/Vietnamese) is consistent with how persons who are foreign-born and/or of older age tend to have limited English proficiency [33]. The sample size did not allow a robust multivariate analysis to tease apart the relative effects of age, income, language preference, or other characteristics.

To improve measurement quality and interpretation of the intervention’s impact overall and by demographic groups, we constructed three summary measures using all KAB items asked at both baseline (pre-intervention) and following intervention (post-test). Leveraging the rich questionnaire data and the Likert type of responses, the continuous summary measures allowed for improved precision. However, we did not measure understanding of genetic testing directly and such a knowledge-based item may be included in future research. Also, the examples in the genetic testing items could have led to higher scores than if the examples had been related to mental health or psychiatric conditions, but that taken together, the difference between the pre- and post- genetic testing scores and the increase in the mental health and help-seeking scores suggest that the overall effects would have been similar if genetic testing examples had included mental health conditions. In addition, continuous outcome measures allow for deeper investigation into sample variability, a marked improvement to more traditional *p*-value pursuits.

## 5. Conclusions

This study demonstrates the feasibility of K-drama as an innovative and widely available health educational tool to educate communities about precision mental health. K-dramas may play an important role in affecting population-level change globally in diverse health issues as well as promoting health equity among vulnerable populations.

## Figures and Tables

**Table 1:** Sample Characteristics, Overall and By Workshop Participation (*n* = 122).

Characteristics	All	Workshop Participation by Language			
		English * (*n* = 36)	Korean (*n* = 45)	Vietnamese (*n* = 41)	*p*-Value
**Gender (%)**					0.936
Female	71.3	69.4	71.1	73.2	
Male	28.7	30.6	28.9	26.8	
**Mean Age (years) at recruitment, (SE)**	100	29.3 (15.8)	67.4 (19.9)	51.9 (15.9)	<0.001
**Age Group (years)**					<0.001
<35	32.8	83.3	2.2	22.0	
35–64	35.2	11.1	37.8	53.7	
≥65	32.0	5.6	60.0	24.4	
**Current marital status (%)**					<0.001
Single	29.5	72.2	8.9	14.6	
Married/Living together	50.8	13.9	60.0	73.2	
Separated/Divorced/Widowed	19.7	13.9	31.1	12.2	
**Education (%)**					0.003
Less than high school	10.7	0.0	11.1	19.5	
High school	17.2	19.4	15.6	17.1	
Some college	28.7	33.3	13.3	41.5	
College or more	43.4	47.2	60.0	22.0	
**Employment status (%)**					<0.001
Full time	15.6	22.2	0.0	26.8	
Part time	23.8	36.1	8.9	29.3	
Homemaker	23.7	5.6	48.9	12.2	
Not looking	21.3	2.8	40.0	17.1	
Disabled/Retired	10.6	22.2	0.0	12.2	
Other/Unknown	4.9	11.1	2.2	2.4	
Household income (%) **					<0.001
<$25,000	42.6	19.4	48.9	56.1	
$25,000–75,000	27.9	44.4	11.1	31.7	
$75,001–150,000	15.5	22.2	13.3	12.2	
>$150,000	11.5	8.3	24.4	0.0	
**Nativity (%)**					<0.001
US-born	19.7	63.9	0.0	2.4	
Foreign-born	80.3	33.1	100.0	97.6	
Number of years watching K-dramas (%) **					0.316
≤1	17.2	11.1	8.9	9.5	
>1	82.8	88.9	91.1	80.5	
**Currently watches K-dramas**					0.052
Yes	69.7	66.7	82.2	58.5	
No	30.3	33,3	17.8	41.5	
Number of hours per week watching K-dramas **					0.085
≤1	18.9	16.7	21.6	16.7	
2–5	43.5	29.2	45.9	54.2	
≥5	23.5	20.8	24.3	25.0	
Varies	14.1	33.3	8.1	4.2	
Enjoy watching K-dramas **					0.677
Yes	76.9	83.3	73.3	75.0	
No	16.5	13.9	20.0	15.0	
Not sure	6.6	2.8	6.7	10.0	

Notes: * The English-speaking participants included Chinese (including Taiwanese) *n* = 7, Filipino *n* = 3, Japanese *n* = 4, Burmese *n* = 1, Vietnamese, *n* = 4, Korean *n* = 3, Mixed *n* = 7, and “Asian” unspecified, *n* = 6. ** Household income does not add to 122 as three participants declined to report it. For number of hours per week watching K-dramas, the *n* was based on their affirmative (“Yes”) response to “currently watches K-dramas” (*n* = 85) There was one participant who did not respond to the question about whether they “Enjoy watching K-dramas”.

**Table 2:** Unweighted and Weighted Pre- and Post-Summary Scores Overall and for Genetic Testing, Mental Health and Health-Seeking (*n* = 118) *.

Survey	Items	Pre-Score	Post-Score	Mean Change	95% CI
Overall, unweighted	16	3.99	4.18	0.193	0.131, 0.256
Overall, weighted	16	4.00	4.18	0.191	0.129, 0.253
Genetic testing	7	3.90	4.09	0.194	0.111, 0.283
Mental health and help-seeking	9	4.08	4.27	0.189	0.114, 0.264

* Overall weighted takes into account that the mental health and help-seeking component had two more items than the genetic test component (weighted towards mental health), while the unweighted approaches considers each subscale as equal in contribution to the overall score.

**Table 3:** Pre- and Post-Difference in Summary Scores by Socio-demographic Characteristics: Overall, Genetics and Genetic Testing, and Mental Health and Help-Seeking (*n* = 118).

Category	Overall Change in Score	Change in Genetics and Genetic Testing Score	Change in Mental Health and Help-Seeking Score
**Sex**			
Female (*n* = 83)	0.18 (0.10, 0.25) *	0.17 (0.07, 0.27) *	0.19 (0.10, 0.28) *
Male (*n* = 35)	0.23 (0.12, 0.35) *	0.27 (0.09, 0.45) *	0.19 (0.07, 0.34) *
**Age Group (in Years)**			
<35 (*n* = 39)	0.23 (0.14, 0.32) *	0.12 (0.03, 0.23) *	0.34 (0.22, 0.45) *
35–64 (*n* = 43)	0.16 (0.06, 0.25) *	0.16 (0.02, 0.29) *	0.16 (0.04, 0.29) *
≥65 (*n* = 36)	0.20 (0.08, 0.33) *	0.33 (0.16, 0.51) *	0.07 (−0.06, 0.19)
**Marital Status**			
Married/living together (*n* = 61)	0.12 (0.06, 0.20) *	0.15 (0.04, 0.26) *	0.09 (−0.02, 0.19)
Single (*n* = 36)	0.27 (0.16, 0.38) *	0.19 (0.07, 0.31) *	0.34 (0.22, 0.46) *
Separated/Divorced/Widowed (*n* = 21)	0.27 (0.11,0.44) *	0.34 (0.11, 0.60) *	0.21 (0.04, 0.37) *
**Employment Status**			
Full time (*n* = 19)	0.13 (0.01, 0.25) *	0.01 (−0.03, 0.22)	0.17 (0.01, 0.34) *
Homemaker (*n* = 26)	0.09 (−0.03, 0.23)	0.11 (−0.07, 0.29)	0.08 (−0.10, 0.24)
Not looking (*n* = 26)	0.16 (0.04, 0.30) *	0.30 (0.07, 0.53) *	0.01 (−0.08, 0.13)
Part time (*n* = 28)	0.25 (0.15, 0.36) *	0.18 (0.05, 0.32) *	0.32 (0.19, 0.46) *
Other/Unknown (*n* = 19)	0.36 (0.19, 0.53) *	0.31 (0.11, 0.50) *	0.41 (0.21, 0.60) *
**Household Income**			
<$25K (*n* = 49)	0.20 (0.10, 0.30) *	0.27 (0.13, 0.43) *	0.12 (0.02, 0.22) *
$25–75K (*n* = 34)	0.23 (0.11, 0.35) *	0.22 (0.09, 0.37) *	0.24 (0.10, 0.38) *
$75–150K (*n* = 18)	0.15 (0.00, 031) *	0.08 (−0.11, 0.27)	0.23 (0.05, 0.39) *
>$150K (*n* = 14)	0.12 ( −0.04, 0.28)	−0.01 (−0.22, 0.22)	0.25 (0.04, 0.45) *
**Workshop Participation**			
English (*n* = 36)	0.29 (0.17, 0.39) *	0.18 (0.07, 0.29) *	0.39 (0.28, 0.50) *
Korean (*n* = 42)	0.11 (0.01, 0.21) *	0.18 (0.01, 0.36) *	0.04 (−0.07, 0.14)
Vietnamese (*n* = 40)	0.20 (0.09, 0.31) *	0.23 (0.10, 0.36) *	0.17 (0.03, 0.30) *
**Educational Attainment**			
High school or less (*n* = 30)	0.28 (0.17, 0.40) *	0.35 (0.20, 0.50) *	0.21 (0.08, 0.34) *
Some college (*n* = 35)	0.18 (0.07, 0.29) *	0.18 (0.05, 0.33) *	0.18 (0.04, 0.33) *
College (*n* = 53)	0.15 (0.05, 0.24) *	0.12 (−0.01, 0.27)	0.18 (0.07, 0.30) *

Mean scores reported on summary measures. Not standardized as all original items originated from equivalent 5-point ordinal scale. ^*^ *p* ≤ 0.05. Not adjusted for multiple testing. Household income does not add to 118, as 3 persons did not respond.

**Table 4:** Perspectives about the Utility of K-drama as a Precision Mental Health Educational Tool (*n* = 122).

	Mean Score (SD)				
Korean Dramas Can ...	All	Workshop Participation by Language			
		English (*n* = 36)	Korean (*n* = 45)	Vietnamese (*n* = 41)	*p*-Value
1. Help community to understand how genetics affects health	4.22 (0.64)	4.25 (0.84)	4.05 (0.49)	4.39 (0.54)	0.046
2. Help community to get genetic testing	4.09 (0.73)	3.94 (0.91)	3.92 (0.62)	4.39 (0.59)	0.005
3. Help community to understand what mental health is	4.28 (0.62)	4.39 (0.69)	4.00 (0.53)	4.49 (0.55)	<0.001
4. Help community to seek help for mental health problems	4.24 (0.63)	4.36 (0.76)	4.00 (0.48)	4.39 (0.59)	0.006
5. Satisfied with the workshop	4.50 (0.64)	4.50 (0.85)	4.36 (0.53)	4.65 (0.48)	0.119
